# Balancing Act: Tubulin Glutamylation and Microtubule Dynamics in *Toxoplasma gondii*

**DOI:** 10.3390/microorganisms12030488

**Published:** 2024-02-28

**Authors:** Inês L. S. Delgado, João Gonçalves, Rita Fernandes, Sara Zúquete, Afonso P. Basto, Alexandre Leitão, Helena Soares, Sofia Nolasco

**Affiliations:** 1 CIISA—Centro de Investigação Interdisciplinar em Sanidade Animal, Faculdade de Medicina Veterinária, Universidade de Lisboa, 1300-477 Lisboa, Portugal; ines.delgado@ulusofona.pt (I.L.S.D.); ritamfernandes323@gmail.com (R.F.); olivesara18@gmail.com (S.Z.); abasto@fmv.ulisboa.pt (A.P.B.); alexandre@fmv.ulisboa.pt (A.L.); 2 Laboratório Associado para Ciência Animal e Veterinária (AL4AnimalS), 1300-477 Lisboa, Portugal; 3 Faculdade de Medicina Veterinária, Universidade Lusófona—Centro Universitário de Lisboa, 1749-024 Lisboa, Portugal; 4 Evotec, Campus Curie 195 Route d’Espagne, 31036 Toulouse, France; joao.alg@gmail.com; 5 Escola Superior de Tecnologia da Saúde de Lisboa, Instituto Politécnico de Lisboa, 1990-096 Lisboa, Portugal; helena.soares@estesl.ipl.pt; 6 Centro de Química Estrutural, Institute of Molecular Sciences, Faculdade de Ciências da Universidade de Lisboa, 1749-016 Lisboa, Portugal

**Keywords:** *Toxoplasma gondii*, tubulin glutamylation, tubulin post-translational modifications, apical complex, microtubules

## Abstract

The success of the intracellular parasite *Toxoplasma gondii* in invading host cells relies on the apical complex, a specialized microtubule cytoskeleton structure associated with secretory organelles. The *T. gondii* genome encodes three isoforms of both α- and β-tubulin, which undergo specific post-translational modifications (PTMs), altering the biochemical and biophysical proprieties of microtubules and modulating their interaction with associated proteins. Tubulin PTMs represent a powerful and evolutionarily conserved mechanism for generating tubulin diversity, forming a biochemical ‘tubulin code’ interpretable by microtubule-interacting factors. *T. gondii* exhibits various tubulin PTMs, including α-tubulin acetylation, α-tubulin detyrosination, Δ5α-tubulin, Δ2α-tubulin, α- and β-tubulin polyglutamylation, and α- and β-tubulin methylation. Tubulin glutamylation emerges as a key player in microtubule remodeling in *Toxoplasma*, regulating stability, dynamics, interaction with motor proteins, and severing enzymes. The balance of tubulin glutamylation is maintained through the coordinated action of polyglutamylases and deglutamylating enzymes. This work reviews and discusses current knowledge on *T. gondii* tubulin glutamylation. Through in silico identification of protein orthologs, we update the recognition of putative proteins related to glutamylation, contributing to a deeper understanding of its role in *T. gondii* biology.

## 1. Introduction

The phylum Apicomplexa compromises around 5000 obligate intracellular parasites, many of which are highly significant in veterinary and medical contexts [1]. Key members include species in the genus *Plasmodium*, responsible for malaria in humans, a disease with severe consequences [2]; *Eimeria*, a pathogen affecting poultry and cattle [3,4]; *Cryptosporidium*, an opportunistic pathogen affecting both humans and animals [5]; as well as *Besnoitia*, *Babesia*, and *Theileria*, parasites impacting cattle [6,7,8]. The tissue-cyst-forming coccidian *Toxoplasma gondii* affects both domestic and wild animals [9]. In humans, it commonly causes congenital neurological and ocular defects [10], and poses a serious threat to immunocompromised individuals [11]. Its ability to spread through water and food has led to its categorization as a category B priority pathogen by the National Institute for Allergy and Infectious Diseases (NIAID) [12].

Understanding how parasites enter host cells and proliferate is crucial for comprehending diseases and may aid in identifying targets for the development of novel therapeutic approaches. The invasion process of Apicomplexa zoites and the molecular mechanisms underlying it appear to be conserved. To penetrate host cells, apicomplexans employ a system of adhesion-based motility known as gliding, which has been observed to depend on actin/myosin interactions [13]. In this process, the apical complex, a microtubule (MT)-based cytoskeletal structure localized in the anterior region of the cell, plays a pivotal role in the interaction with the host cell [14]. Importantly, the molecular composition of this structure remains incompletely understood, but it is likely enriched in proteins involved in MT assembly and dynamics, as well as in proteins participating in MT-associated processes and their interplay with other cellular systems (e.g., actin, vesicles). As a crucial component of the apical complex, comprehending how this specialized class of MTs is assembled, maintained, and functionally interacts with other cellular structures is paramount. Current understanding of the biology of the apical complex suggests that tubulin post-translational modifications (PTMs) and the machineries responsible for their generation and removal play pivotal roles in the assembly and functions of this structure during host cell invasion.

## 2. Microtubule Cytoskeleton

Throughout evolution, eukaryotic cells developed highly sophisticated and specialized cytoskeleton systems, including intermediate filaments, actin filaments, and MTs. Despite their specific roles, these structures crosstalk and cooperate, such as in supporting membrane structures like nuclear and plasma membranes, thereby imparting shape and mechanical resistance to the cell [15]. Moreover, eukaryotic cytoskeletons are involved in various processes, including cytoplasmic organization, organelle assembly and maintenance, cell division, cell polarity, cell migration, intracellular transport, and cell signaling [16,17]. In multicellular organisms, the cytoskeleton also plays crucial roles in establishing cell–cell contacts and cell–extracellular matrix interactions, thereby contributing to tissue integrity [18].

MTs are dynamic polymers composed of heterodimers of the structurally and functionally conserved α- and β-tubulins, both of which are GTP-binding proteins and are ubiquitous across all studied eukaryotes [19]. Higher organisms, including humans and mice, possess extensive gene families encoding multiple α- and β-tubulin isotypes [20]. Significantly, certain tubulin isotypes are expressed in a tissue-specific manner, playing key roles in the assembly of specialized functional classes of MTs. For instance, the proper expression of specific tubulin isotypes (e.g., β3-tubulin) is essential for neuronal differentiation and survival in mammals, and mutations in their coding genes are linked to neuronal disorders [21,22,23]. In contrast, lower eukaryotes such as *Saccharomyces* [24,25] and *Tetrahymena* [26,27] typically harbor one or two α- and two β- canonical tubulin-coding genes.

For the assembly of the α/β-tubulin heterodimer, tubulins undergo a complex folding process assisted by molecular chaperones (prefoldin and CCT) [28,29,30,31] and tubulin cofactors (TBCA-E) [32]. Besides their roles in heterodimer assembly, tubulin cofactors also contribute to quality control and recycling of heterodimers released from depolymerized MTs. Thus, by regulating the pool of free tubulin dimers competent to polymerize, the tubulin folding pathway controls MT dynamics [33,34,35,36,37]. Once folded and assembled, tubulin heterodimers polymerize in a polarized head-to-tail manner to form protofilaments, which then assemble into the characteristic hollow structure of MTs, typically composed of 13 protofilaments [19]. During the polymerization process, β-tubulin hydrolyses its GTP to GDP, and upon MT depolymerization, the GDP is exchanged to GTP to enable β-tubulin polymerization again. In contrast, GTP bound to α-tubulin remains not hydrolyzed during polymerization [38,39].

Due to the polar nature of MTs, one end (the minus end) is comprised of α-tubulin subunits, while the other end (the plus end) consists of β-tubulin. Furthermore, the two ends of MTs exhibit distinct dynamic properties, with the minus end presenting slow growth and the plus end undergoing rapid polymerization [19]. Typically, the minus end is associated with MT organizing centers (MTOCs), such as spindle pole bodies in fungi and centrosomes and the Golgi apparatus in animal cells. MTOCs exhibit structural variability across different eukaryotic groups but are consistently enriched in proteins that facilitate MT nucleation (e.g., gamma-tubulin) and anchoring [40,41,42,43].

MTs can present varied dynamic properties and stability, which are influenced by factors such as the preferential incorporation of specific tubulin isotypes (products of different tubulin genes) and by tubulin PTMs such as acetylation, detyrosination, glutamylation, and glycylation [44,45]. Tubulin PTMs can selectively and reversibly affect distinct MT subpopulations [46]. These modifications are evolutionarily conserved and contribute to what is termed the “tubulin code”.

For instance, α-tubulin can undergo acetylation at K40, which is the sole known PTM that localizes in the luminal surface of MTs [44,45]. Additionally, its C-terminal tail can undergo a reversible modification through the deletion of the terminal tyrosine (detyrosination) or an irreversible modification by deletion of the last two residues (Δ2). The C-terminal tails of both α- and β-tubulins can also be reversibly modified by glutamylation and glycylation [44,45]. While the K40 PTM has been linked to MT stability [47], C-terminal PTMs alter MT interactions with associated proteins, thereby influencing sensitivity to MT-targeting drugs.

Tubulin PTMs play a crucial role in the binding of MT-associated proteins (MAPs), such as MT motors and MT-severing proteins, to MTs. Consequently, they are essential for the assembly and maintenance of MT-based organelles such as centrioles, cilia, and flagella, as well as MT cortical structures found in unicellular organisms like *Tetrahymena thermophila* and *T. gondii* [46,48]. Thus, centriolar and axonemal MTs exhibit high levels of acetylation and glutamylation compared to cytoplasmic MTs [49]. In cilia, although the precise impact of tubulin PTMs on intra-flagellar transport is not fully understood, tubulin glutamylation has been shown to affect intra-flagellar transport velocity [50] and the localization of MT motor proteins [51]. Importantly, tubulin PTMs can be regulated in response to environmental cues. In *Caenorhabditis elegans*, for instance, tubulin glutamylation was shown to be upregulated in sensory cilia in response to changes in temperature, osmolality, and dietary conditions [50].

## 3. The Specialized Microtubule Structures of *Toxoplasma gondii*

**Figure 1:**
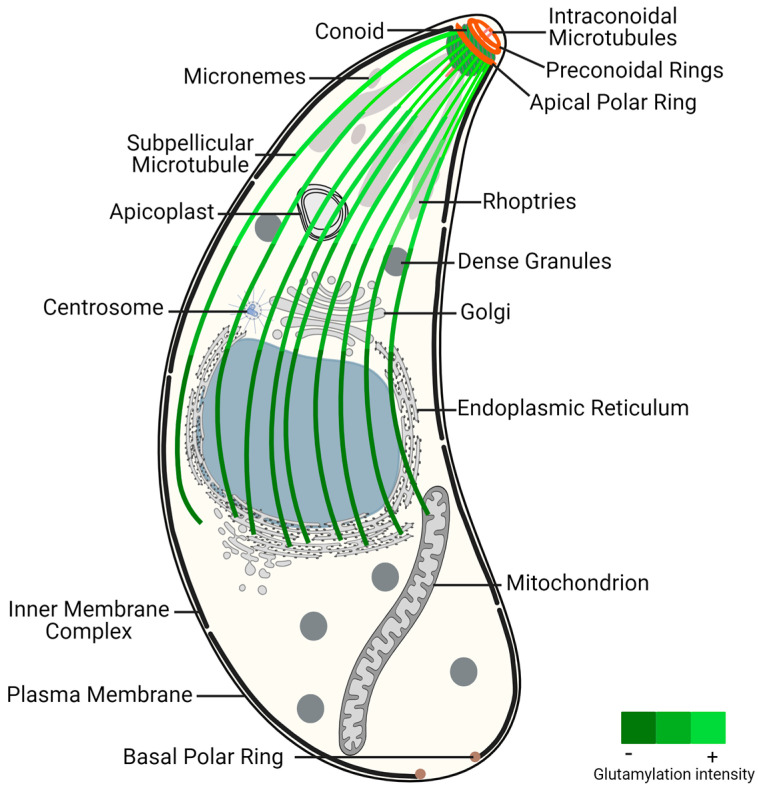
*Toxoplasma gondii* tachyzoite ultrastructure, highlighting the glutamylation of subpellicular microtubules. This post-translational modification presents increased density near the conoid region, an apical structure critical for parasite invasion and motility, decreasing toward the distal end of subpellicular microtubules. The gradient of glutamylation suggests a functional stratification within the microtubule network, essential for the parasite’s life cycle and pathogenicity. Figure generated with BioRender.

Apicomplexans are classified as alveolate organisms due to the presence of a flattened vesicle system (alveoli) underlying their plasma membrane, forming a structure known as the pellicle [52,53,54]. The pellicle is further divided into three subdomains (apical, central, and basal), each with distinct properties conferred by specific cytoskeleton components. The plasma membrane associated with alveoli form the inner membrane complex (IMC), which extends from the apical polar ring (APR) to the basal pole, leaving the extreme apical region of the parasite solely enclosed by plasma membrane. While γ-tubulin does not localize to the APR [55], this structure is regarded as an MTOC because the minus ends of subpellicular MTs (SPMTs) are anchored there via cogwheel-like projections, with their plus ends extending distally from this structure [56,57]. SPMTs extend in a gentle spiral from the APR to a region posterior to the nucleus, contributing to the elongated shape, rigidity, and the maintenance of the highly polarized cell organization [58] (Figure 1). These SPMTs are coated with MAPs [59], among which are Subpellicular Microtubule Proteins 1 and 2 (SPM1 and SPM2), unique to apicomplexan parasites [60]. Up to this point, the proteins identified as components of the APR include TgRNG1, TgRNG2, TgAPR1, and TgKinesin A [56,61,62,63]. TgRNG1 becomes detectable at the mature APR only after nuclear division is complete [61]. TgAPR1 is also a marker of the mature APR structure, and parasites lacking TgAPR1 demonstrate a defect in the lytic cycle [56]. The MT motor TgKinesin A, while not essential, plays a role in parasite growth, and parasites lacking this protein exhibit a modest reduction in growth rate. TgKinesin A localizes to emerging daughter buds and is positioned just apical to APR1 at the APR of mature parasites [56].

The apical complex is centered around the extensible and retractable conoid, which exhibits active extrusion during host invasion [64]. The conoid, mainly composed of tubulin, adopts a unique polymer form distinct from typical MTs [65]. It comprises two preconoidal rings above the conoid and two intraconoidal MTs [66]. While related alveolates may possess incomplete conoids or pseudoconoids, most apicomplexans were thought to have lost the conoid structure, with coccidian parasites, like *T. gondii*, retaining a closed conoid [67]. However, recent data indicate that the conoid is a hallmark of invasion mechanisms conserved in all apicomplexans and is also present in other alveolates [68]. Proteomics analysis of the *T. gondii* conoid/apical complex has identified approximately 200 proteins, representing 70% of *T. gondii* cytoskeleton proteins. These proteins include several key cytoskeletal components such as actin and actin-binding proteins, varied myosin heavy and light chains, and all three isoforms of β-tubulin [14].

At the apical pole, specialized secretory organelles called rhoptries and micronemes play crucial roles traversing within the conoid to secrete their contents across the plasma membrane at the apical tip of the parasite [69].

As part of the MT cytoskeleton, *T. gondii* tachyzoites also possess centrioles, which are barrel-shaped structures formed by nine singlet MTs [58]. Unlike non-coccidian apicomplexans such as *Plasmodium* which lack asexual centrioles, this structure occurs in other coccidians. Coccidian centrioles are relatively short and arranged in a parallel rather than orthogonal configuration [67]. Despite the absence of pericentrin and ninein genes, the term “centrosome” has been used in *T. gondii* due to its nucleating activity and its ability to function as a signaling platform [70].

The nuclear division in *T. gondii* relies on the centrocone, a domain of the nuclear envelope, and occurs without the nuclear envelope breaking down [71,72]. Unlike in other organisms, chromosome segregation in *T. gondii* occurs without chromosome condensation. Spindle MTs originate in the cytoplasm and traverse the nuclear pores of the centrocone. These MTs are crucial for linking centrosomes with the centrocone and for segregating chromosomes into daughter nuclei [71,72]. EB1 proteins bind to the positive ends of dynamic MTs, promoting stability and elongation of the MTs. In *T. gondii*, the EB1 homolog is a nuclear protein that localizes to the centrocone after spindle assembly [73]. Moreover, in addition to the nuclear protein remaining at the centrocone until cytokinesis, there exists a small pool of cytoplasmic TgEB1 that transiently associates with the tips of the daughter buds’ SPMTs after nuclear division is complete [73]. Studies in *T. gondii* have revealed that SPMTs continue to polymerize during daughter cell assembly. However, during later stages of cell division, SPMTs exhibit high resistance to various conditions that typically lead to MT depolymerization, such as cold, antimitotic agents, detergents, and high pressure [58].

MAPs play a critical role in influencing MT stability and imparting different properties to MT populations. While MT motors (dyneins and kinesins), centriole components (SAS-6, centrin, CEP250), and regulatory proteins (EB1) are highly conserved among eukaryotes, proteins associated with SPMTs and the conoid are predominantly specific to these organisms, reflecting the specialized functions of these structures (Morrissette and Gubbels, 2020 [67]). SPMTs are extensively coated with MAPs such as TgSPM1, TgSPM2, TgTrxL1, TgTrxL2, TgTLAP1, TgTLAP2, TgTLAP3, and TgTLAP4. While some of these proteins are distributed throughout SPMTs, others localize to specific subregions [58].

At the basal end, *T. gondii* features a basal complex, devoid of tubulin, which is responsible for completing cytokinesis and thereby facilitating parasite replication [74,75,76].

## 4. *T. gondii* Tubulin Post-Translational Modifications

The *T. gondii* genome harbors genes for three α-tubulin isotypes (α1—TGME49_316400, α2—TGME49_231770, and α3—TGME49_231400) and three β-tubulin isotypes (β1—TGME49_266960, β2—TGME49_221620, and β3—TGME49_212240) [77,78,79]. According to a genome-wide CRISPR screen, α1-, α2-, β1-, and β2-tubulins are essential for in vitro tachyzoites, whereas α3- and β3-tubulins are not [80]. The amino acid sequences of the three β-tubulin isotypes exhibit 96.4–96.9% identity and 98.0–98.7% similarity, with most differences affecting seven of the last eight amino acid residues. Concerning the amino acid sequences of the three α-tubulin isotypes, they present 35.5–68.3% identity and 52.7–79.4% similarity. Notably, among the three α-tubulin isotypes, the α3 isotype exhibits significantly lower percent identity/similarity compared with the other two isotypes. Peptides compatible with the three α- and the three β-tubulin isotypes have previously been detected in *T. gondii* proteomes (evidence available at www.ToxoDB.org, Proteomics section, accessed on 29 September 2023). Of note, the α3-tubulin isotype, the most divergent among the tubulin variants, has only been detected in proteomes analyzing the tachyzoite stage, whereas the other five α- and β-tubulin isotypes have been identified in *T. gondii* proteomes analyzing the tachyzoite, bradyzoite, and oocyst stages (available at www.ToxoDB.org, Proteomics section, accessed on 29 September 2023). The differences in amino acid sequences among the α- and the β-tubulin isotypes are associated with specialized functions and differential expression throughout the *T. gondii* life cycle [78]. Some of these distinct features may be linked to specialized tubulin structures, such as the conoid and the flagellar axoneme [78]. Similar to other eukaryotes, the most divergent region in the amino acid sequences of the different *T. gondii* tubulin isotypes is localized at their C-terminal ends [77,79]. This region becomes exposed on the outer surface of MTs when tubulin dimers polymerize [81], providing binding sites for several MAPs and molecular motors [82]. Additionally, tubulin C-terminal ends undergo various PTMs, with most occurring after tubulin subunits polymerize into MTs. Therefore, distinct C-terminal amino acid sequences likely indicate different patterns of tubulin PTMs and unique associations with MAPs and motor proteins. PTMs identified in *T. gondii* tubulins include acetylation, detyrosination, truncation, methylation, and polyglutamylation [48,83,84,85].

Independent studies described α-tubulin acetylation at the lysine K40 [48,83]. This acetylation, catalyzed by an α-tubulin acetyltransferase (ATAT), is crucial for completing nuclear division, and acetylated α-tubulin is notably enriched during daughter bud formation [83].

Several sources also reported the removal of the last C-terminal amino acid residue Y453, resulting in detyrosinated tubulin [48,83,85]. Antibody detection of detyrosinated tubulin has shown its diffuse presence in SPMTs with an apparent accumulation at their posterior end [48,85].

*T. gondii* α-tubulin has also been reported in C-terminal truncated forms, namely Δ2α-tubulin and Δ5α-tubulin, lacking the two or five most C-terminal residues, respectively [48,83,85]. An antibody targeting mammalian Δ2α-tubulin labeled the apical region of *T. gondii* [48,85].

Moreover, methylation has been reported on α1- and β1-tubulin, a PTM not previously described in tubulins of other organisms, suggesting it might be a specific modification in Apicomplexa [48].

Conversely, glycylation, a modification specific to ciliated cells and enriched in axonemes and basal bodies, was not observed, consistent with the absence of flagellar structures in the *T. gondii* tachyzoite stage.

Despite these findings, a comprehensive understanding of the association between the various PTMs and their functional impact on the parasite’s life cycle is still lacking. In *T. gondii*, secretion plays a crucial role in the successful invasion of host cells. Research has demonstrated that vesicles, within epithelial cells, traverse from the trans-Golgi network to the plasma membrane via polyglutamylated MTs [86], suggesting a connection between tubulin glutamylation and vesicle transport. These findings propose that polyglutamylated MTs may function as a “fast track” in vesicle transport. Indeed, as mentioned above, tubulin glutamylation in cilia impacts the speed of transport and the localization of MT motor proteins [50,51]. Importantly, ciliates and apicomplexans present differences in PTM-meditated tubulin regulation, which may be specific to each type of unicellular organism. Thus, gaining a deeper understanding of the regulation of tubulin glutamylation in *T. gondii* is imperative.

## 5. *T. gondii* Tubulin Glutamylation

**Figure 2:**
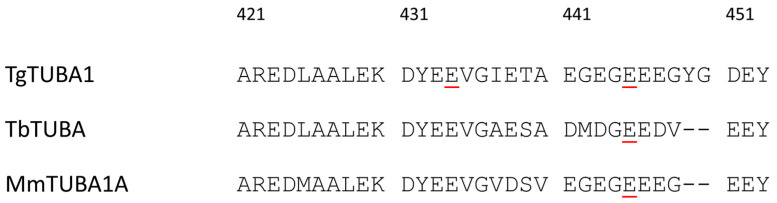
Protein sequence alignment and comparison of the C-terminal end of α-tubulins. Sequences represent *T. gondii* α1-tubulin (TgTUBA1, TGME49_316400), *Trypanossoma brucei* α-tubulin (TbTUBA, Tb1125.1.2340), and *Mus musculus* α1a-tubulin (MnTUBA1A, P68369). Residues with red underline have been documented to be glutamylated [48,88,91]. Numbers on top represent the residue number of *T. gondii* α1-tubulin. Alignment performed using MultAlin [96].

In *T. gondii*, polyglutamylation was detected on both α- and β-tubulins [48,85,87]. Plessman et al. (2004) detected polyglutamylation of α-tubulin at the glutamate residue E445, with glutamate chains containing up to three residues [85] (Figure 2). Notably, glutamylation has also been detected at the α-tubulin E445 residue in mammals, namely mouse, rat, and pig, as well as in the kinetoplastids *Trypanosoma brucei*, indicating a high degree of conservation of this PTM at this residue [88,89,90,91]. Additionally, Xiao et al. (2010) found polyglutamylation of tubulin isotypes α1, β1, and β2, with glutamate chains containing up to four, six, and three residues, respectively [48]. Of note, in this study, glutamate chains detected in α1-tubulin were located at E434, a different residue from previous reports [48], suggesting that tubulin polyglutamylation in *T. gondii* occurs at multiple residues (Figure 2). Multiple glutamylated residues have also been reported in the ciliate *Tritrichomonas mobilensis* [92] and in the rat α4-tubulin isotype [90], although this PTM is often found at a single residue [93,94]. Interestingly, the α1-tubulin E434 residue can undergo both glutamylation and methylation, indicating potential competition for the same residue between the two PTMs, thus influencing each other. This feature parallels findings in *T. thermophila*, where tubulin molecules can be simultaneously glycylated and glutamylated, with the levels of each PTM being related [95].

**Figure 3:**
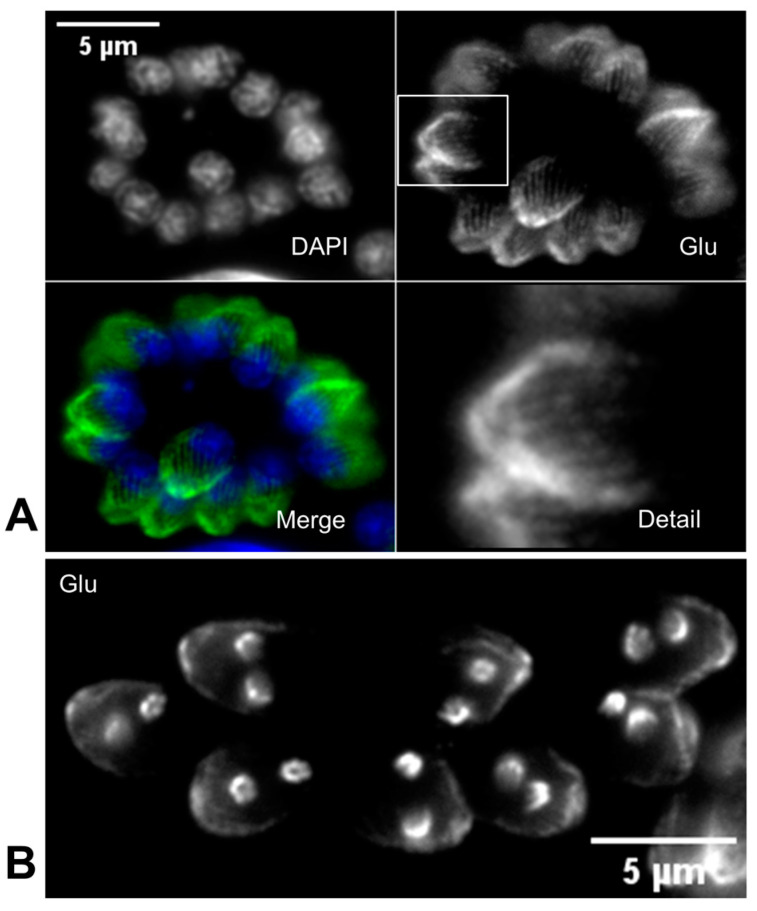
Tubulin glutamylation in *T. gondii*. *T. gondii* tachyzoites stained with anti-polyglutamylation modification GT335 antibody (Glu) detecting glutamylated tubulin. (**A**) Subpellicular microtubules are highly glutamylated, with the highest levels of glutamylation at the apical pole near the conoid and decreasing toward the basal pole. (**B**) Glutamylation is also visible in the forming apical complex of the two daughter cells during tachyzoite endodyogeny. Nucleic acid was stained with 4,6-diamidino-2-phenylindole (DAPI). Scale bars represent 5 µm.

In *T. gondii*, the GT335 antibody, targeting glutamylated tubulin, reveals a gradient of glutamylation near the conoid, decreasing towards the distal end of the MTs (Figure 3A). Similar gradients have been observed along axonemal MTs in various species’ cilia [85,87]. Interestingly, it has been proposed that the apical complex originated from a repurposed flagellum [97]. Glutamylated tubulin, like acetylated α-tubulin [83], is enriched during the formation of daughter buds (Figure 3B). While GT335 detects all glutamylated proteins by targeting the glutamate side-chain branching point, other antibodies like B3 and polyE specifically target polyglutamylated side chains [98,99]. These antibodies also detect glutamylated tubulin in *T. gondii*, indicating the presence of this PTM in its extended form [48,85,87]. Tosetti et al. (2020) utilized the polyE antibody in ultrastructure expansion microscopy, confirming high levels of polyglutamylation in the SPMTs, except at their distal ends. Additionally, the study reported the absence of polyE staining in the conoid fibers, suggesting that no polyglutamylation occurs in this structure [87].

Tubulin polyglutamylation serves as a direct regulator of MT function. It governs interactions between MTs and dynein, a molecular motor protein [100,101], and long glutamate side chains on tubulin serve as a signal for MT severing by spastin [102]. Importantly, tubulin polyglutamylation is a reversible PTM [103]. In mammalian cells, controlling the length of the polyglutamate side chains on tubulin is critical for neural survival [104].

Polyglutamylation is tightly regulated through a coordinated enzymatic process. Polyglutamylase enzymes are classified under the tubulin tyrosine ligase-like (TTLL) protein family [99], while deglutamylase enzymes belong to the cytosolic carboxipeptidase (CCP) family [103,105]. Maintaining appropriate levels of tubulin polyglutamylation is essential for cellular functions and relies on the balanced activities of polyglutamylating and deglutamylating enzymes.

**Figure 4:**
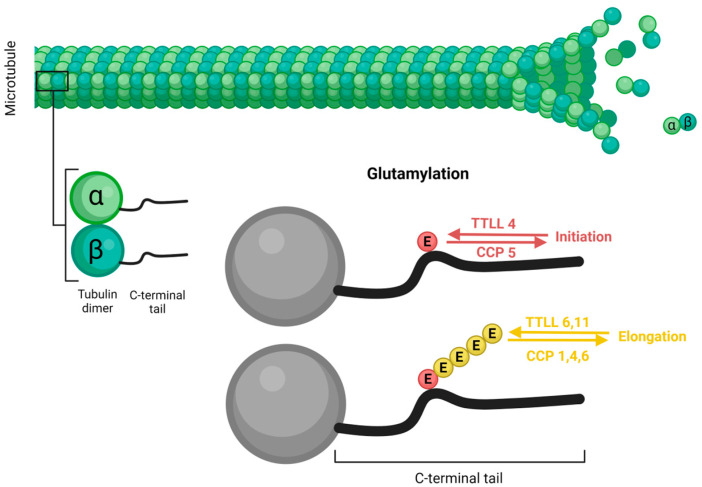
Microtubule glutamylation. Glutamylase enzymes, belonging to the tubulin tyrosine ligase-like (TTLL) protein family, and deglutamylase enzymes, members of the cytosolic carboxipeptidase (CCP) family, are responsible for the addition and removal of glutamate residues (E), respectively, to the carboxy-terminal tail (C-terminal tail) of α- and β-tubulin. TTLL4 initiates glutamylation by adding the first E to the C-terminal tail, which is removed by CCP5. TTLL6 and -11 are responsible for polyglutamylation, elongating the chain by adding additional Es which are removed by CCP1, -4, and -6. Figure generated with BioRender.

The formation of the polymodification side chain occurs through two biochemically distinct steps: initiation and elongation. Often, these steps are mediated by different enzymes within the TTLL family, each exhibiting distinct enzymatic characteristics. For instance, some TTLLs, such as TTLL4, generate short side chains, while others like TTLL6 and TTLL11 add long side chains [99] (Figure 4).

A computational analysis searching for orthologs of proteins associated with glutamylation in *Homo sapiens* and *Tetrahymena thermophila* within the *T. gondii* genome (www.ToxoDb.org, accessed on 29 September 2023) identified six putative genes encoding the TTLLs enzymes, one putative gene encoding a CCP, one putative gene encoding spastin, and one putative gene encoding katanin p60 subunit (Table 1). However, it is crucial to acknowledge that glutamylation can occur in non-tubulin proteins [99], and the same TTLL enzyme can glutamylate both tubulin and non-tubulin substrates [99,106]. Additionally, CCPs are also capable of deglutamylating non-tubulin proteins [104,107].

The observation that only TTLL6 (TGME49_230670) and TTLL11 (TGME49_244500) are predicted to be essential in the *T. gondii* tachyzoite stage [80], while the other TTLL orthologs are non-essential, suggests that TTLL6 and TTLL11 may play crucial roles in regulating polyglutamylation in *T. gondii*. Proteomic analysis supporting the existence of only TTLL11 orthologs further strengthens the hypothesis that TTLL11 enzymes are key players in polyglutamylation regulation.

The presence of two orthologs of mammalian TTLL11 and the absence of TTLLs responsible for short side chains raises the intriguing possibility that one of these orthologs may be involved in producing short glutamylation chains. However, experimental studies are needed to confirm this hypothesis.

The retention of non-essential genes in the *T. gondii* genome, without protein evidence, raises questions about their functional significance. Although these candidate genes lack protein evidence, their transcription suggests they may play regulatory roles. Understanding the functions of these transcripts and whether they indeed play regulatory roles remains an open question that warrants further investigation.

Like TTLLs, CCPs exhibit differences in their enzymatic specificities. Some CCPs, such as CCP1, CCP4, and CCP6, facilitate the shortening of polyglutamate chains, while CCP5 specializes in the removal of branching-point glutamates [103,105] (Figure 4). In addition, CCP1, CCP4, and CCP6 also remove gene-encoded glutamates from the carboxyl termini of proteins, converting detyrosinated tubulin into α2-tubulin [104]. Furthermore, these enzymes demonstrate a high specificity towards the activity of counterparts from the opposing class. For instance, CCP1 is able to shorten the polyglutamate side chains and eliminate branching point glutamates in instances where glutamylation is generated by TTLL6, but not by TTLL4 or by TTLL1 [108]. *T. gondii* possesses a single putative CCP, ortholog of *H. sapiens* CCP1 and *T. thermophila* CCP3, which likely collaborates with the orthologs of TTLL6 and TTLL11. It is a potential candidate for both functions described for CCPs in mammals, namely, the removal of glutamate branching points and shortening of glutamate chains.

Interestingly, tubulin glutamylation has an increasingly well-documented role in the assembly and function of complex microtubular organelles, like cilia, in which tubulin polyglutamylation is observable [46]. Additionally, tubulin glutamylation can also destabilize MTs by regulating MT-severing factors like katanin [109] and spastin [102]. Katanin and spastin belong to closely related MT-severing enzyme families that are widely distributed throughout eukaryotes [110]. MT severing involves generating an internal break in an MT, potentially increasing polymer mass by producing shorter MTs that can serve as seeds for nucleating new ones. Generally, both severing enzymes are much more activated by long glutamate side chains than by short side chains. However, they exhibit different activation patterns in response to specific TTLLs; TTLL11 has a weaker impact on katanin activation compared to its strong effect on spastin-mediated severing. Thus, although spastin is insensitive to these differences, katanin is more efficiently activated by glutamylation generated by TTLL6. This suggests that fine-tuning these side chains could have an implication on the differential activation of these proteins [102].

In *T. gondii*, although we identified a candidate katanin p60 subunit ortholog, this gene is not essential, lacking protein evidence from proteomic analysis. However, no ortholog candidate for the katanin p80 subunit was identified, suggesting that there is no katanin-like functional protein. On the other hand, there is a strong candidate spastin ortholog, an essential gene that presents protein evidence. These data suggest that the spastin ortholog is likely the enzyme responsible for MT severing in *T. gondii*. Interestingly, mammalian TTLL11 triggers a strong MT severing response by spastin [102], suggesting that the two activities may operate together in *T. gondii* to modulate MT structures’ remodeling and dynamics.

While *T. gondii* exhibits specific and intricate MT structures such as those found in the conoid, the polyglutamylation process is likely less complex than in human cells. This distinction may arise from more structural functions leading to more stable MTs in *T. gondii*, whereas the greater tubulin glutamylation complexity in mammals may be associated with more regulatory functions, resulting in more dynamic MTs across various cell types with distinct functions. However, the generated patterns in conjunction with other PTMs (including specific methylation) are sufficient to ensure distinct features. These patterns influence the interactions with MAPs, impacting functions that may be related to microneme and rhoptry secretion, as well as conoid extension/retraction.

## 6. Concluding Remarks

Polyglutamylation is a strictly regulated tubulin PTM; therefore, to understand its role in MT dynamic regulation in *T. gondii*, it is essential to identify and characterize the enzymes involved in the generation and removal of the glutamate side chains. As expected, this parasite appears to present a smaller set of enzymes involved in polyglutamylation regulation, but still possess the key components of a similar regulation process, expressing enzymes that participate in each step, namely glutamylation, deglutamylation, and microtubule severing responsive to glutamylation. Experimental data are needed to assess and validate the functions of these enzymes in *T. gondii*.

## Figures and Tables

**Table 1:** *T. gondii* orthologs of genes involved in glutamylation.

*T. gondii* Gene			*H. sapiens* Ortholog		*T. thermophila* Ortholog	
ToxoDB ID	Fitness Score ^1^	Protein Evidence ^2^	Designation	Uniprot Reference	Designation	Uniprot Reference
TGME49_278930	non-essential	no	TTLL1	O95922	TTLL9	I7MCG4
TGME49_228410	non-essential	no	TTLL2	Q9BWV7		
TGME49_230670	essential	no	TTLL6	Q8N841		
TGME49_500030	essential	no			TTLL6A	Q23MT7
TGME49_244500	essential	yes	TTLL11	Q8NHH1		
TGME49_307760	non-essential	yes	TTLL11	Q8NHH1		
TGME49_265780	essential	yes	CCP1	Q9UPW5	CCP3	Q23FW3
TGME49_315680	essential	yes	Spastin	Q9UBP0	AAA Family ATPase 1	Q236J5
TGME49_244590	non-essential	no	Katanin p60 subunit	O75449	Katanin1	Q237K9

^1^ Predicted to be essential/non-essential based on the fitness score obtained from a CRISPR-Cas9 genome wide loss of function screen [80], data available at www.ToxoDB.org, Phenotype section. ^2^ Data available at www.ToxoDB.org, Proteomics section.

## Data Availability

No new data were created during this study. The data analyzed referred throughout the text are publicly available at www.ToxoDB.org (accessed on 29 September 2023).
